# Pre-operative imaging evaluation of renal cell carcinoma

**DOI:** 10.1590/1806-9282.2024S107

**Published:** 2024-06-07

**Authors:** Paulo Victor Alves Pinto, Fernando Morbeck Almeida Coelho, Alice Schuch, Mauricio Zapparoli, Ronaldo Hueb Baroni

**Affiliations:** 1 Hospital Israelita Albert Einstein, Brazilian College of Radiology Genitourinary Group, Department of Radiology – São Paulo (SP), Brazil.; 2 Hospital Moinhos de Vento, Brazilian College of Radiology Genitourinary Group, Department of Radiology – Porto Alegre (RS), Brazil.; 3 Advanced Imaging Diagnosis, Brazilian College of Radiology Genitourinary Group, Department of Radiology – Curitiba (PR), Brazil.

## INTRODUCTION

Renal cell carcinoma (RCC) is one of the most common cancers worldwide, with approximately 4,31,288 new cases and 1,79,368 deaths globally in 2020 and 81,800 new cases in the United States alone^1,2^. RCC may present with flank pain, mass, or hematuria, but its incidence has increased due to incidental diagnosis through imaging methods^3^.

The standard treatment for localized RCC includes surgical and imaging-guided non-invasive procedures, such as ablation, nephron-sparing (NS) partial nephrectomy (PN), and radical nephrectomy (RN). Precise preoperative imaging is essential in determining the surgical approach, and imaging methods also play a critical role in subtype characterization and staging. Therefore, adhering to structured and updated guidelines for appropriately utilizing imaging methods in evaluating RCC is crucial^4,5^.

This article aims to provide a comprehensive overview of the preoperative role of imaging, including imaging protocols, epidemiological insights, subtype characterization, staging, and structured reporting in assessing RCC.

## PROTOCOLS

### Computed tomography

#### Overview

Computed tomography (CT) is the most commonly used imaging technique for presurgical planning, detection, and post-therapy monitoring of renal masses. It also plays a significant role in detecting renal lesions incidentally. CT is faster and more readily available than MRI, is less prone to imaging artifacts, and provides better spatial resolution. Compared to ultrasound (US), which is equally functional, CT is less dependent on operator skills. It offers a better view of the perirenal space without bowel gas interposition or patient body fat composition limitations. Intravenous contrast administered during CT scans allows for better characterization of homogeneous masses and more accurate subtype prediction based on the enhancement pattern.

#### Computed tomography protocols

Our CT protocol follows Society of Abdominal Radiology RCC Disease-Focused Panel guidelines for pre-nephrectomy and pre-ablation mass characterization, using a combination of pre- and post-contrast imaging acquisitions (Table 1)^6^.

**Table 1:** Computed tomography and magnetic resonance imaging protocol for evaluation of renal masses.

Contrast-enhanced CT protocol					
	Precontrast phase	Corticomedullary phase	Nephrographic phase	Excretory phase	Technical notes
Iodine contrast vol.	–	1.2 mL/kg	1.2 mL/kg	1.2 mL/kg	- Use a straight-back support - Patient lying flat in a supine position with both arms elevated -Axial laser: intermammillary line -Coronal laser: axillary line Sagittal laser: midline
Contrast flow	–	4 mL/s	4 mL/s	4 mL/s	
Acquisition time	–	40 s	80 s	5 min	
kV	120	120	120	120	
Range	800	800	800	800	
Rot time	0.5	0.5	0.5	0.5	
FOV	Upper abdomen	Upper abdomen	Upper abdomen and pelvis	Upper abdomen	
Pitch	Thickness 0.5×80 (Std)	Thickness 0.5×80 (Std)	Thickness 0.5×80 (Std)	Thickness 0.5×80 (Std)	
Thickness	1.0×0.8 mm	1.0×0.8 mm	1.0×0.8 mm	1.0×0.8 mm	
**Dynamic MRI protocol**					
	**Axial T2WI fat-sat trigger**	**Axial DWI trigger (B400-800)**	**2D coronal T2WI**	**CORONAL 3D pre- and post-contrast**	**Axial 3D-GRE in-out phase**
FOV (cm)	34	34	38	38	30
Thickness/GAP	6/1	6/1	5/1	3.8	5/1
Matrix (frequency/phase)	320	192/224	256/224	256/224	256/160
NEX/224	1.5	3/5	1	1	1
Band	83	250	31.5	83	83

A renal scan includes four phases, namely, precontrast, corticomedullary, nephrographic, and excretory. The precontrast phase helps detect fat, hemorrhagic content, and calcifications. The corticomedullary phase helps map the vasculature and determine lesion enhancement patterns. The nephrographic phase is most effective for detecting renal lesions and identifying poorly vascularized tumors. The excretory phase characterizes the involvement of the renal collecting system and differentiates non-renal cell subtypes such as urothelial carcinoma.

### Magnetic resonance imaging

#### Overview

Magnetic resonance imaging (MRI) is helpful for preoperative renal mass examination. It does not emit ionizing radiation, making it suitable for pregnant women, children, and patients with prior radiation exposure. Gadolinium can replace iodinated contrast, which is beneficial for allergic patients and does not cause kidney damage (although it is contraindicated during pregnancy and linear molecule formulations of gadolinium must be avoided in patients with renal failure).

#### Magnetic resonance imaging protocols

Our institutional protocol for abdominal imaging follows the general MRI guidelines of the Society of Abdominal Radiology RCC Disease-Focused Panel (Table 1)^7^. We use two-dimensional (2D) T2-weighted (T2W) fast spin-echo (FSE) sequences in the axial or coronal planes of the upper abdomen, with and without fat suppression, to characterize macroscopic fat and obtain a general overview of upper abdominal structures. 3D T1-weighted (T1W) gradient-recalled echo (GRE) sequences in in-phase and out-of-phase imaging can help identify microscopic fat and hemorrhagic content, while dynamic 3D T1W fat-suppressed sequences before and after contrast administration can provide information on vascularization, subtype prediction, and renal vasculature. We use diffusion-weighted imaging (DWI) sequences with b-values of 400 and 800 to better detect small renal masses and identify lymph nodes and secondary lesions.

## SUBTYPES AND HISTOLOGICAL PREDICTION

### Epidemiology

The 5th edition of the World Health Organization (WHO) classification of renal tumors has introduced genetics and molecular features for subtype characterization, comprising 20 different entities^5^. While this might help tailor treatment in the future, current guidelines rely on distinguishing between clear-cell RCC (ccRCC), which represents about 75% of the lesions, and non-clear RCC^4^. Most non-clear RCC cases correspond to papillary RCC (pRCC) and chromophobe RCC (chRCC)^8,9^. Identifying the features that suggest specific subtypes, particularly ccRCC, is crucial in the imaging workup. Distinguishing between these entities can speed up the treatment of patients at higher risk and theoretically prevent disease progression or metastasis.

### Clear-cell renal cell carcinoma

Clear-cell renal cell carcinoma is a malignant tumor originating from the renal cortex's tubular epithelial cells. It displays a wide range of morphological variations, making it a prevalent subtype of sporadic RCCs in adults. It is responsible for about 75% of all cases^5,9^. It is more likely to develop in individuals over 60 years old, with a slightly higher occurrence in men and a higher prevalence among white individuals than black individuals.

The clinical behavior of ccRCC is more aggressive than other RCC subtypes and has a higher potential for metastasis, particularly for solid tumors, than those with solid-cystic characteristics. This risk is due to the potential for late-stage diagnosis and its resistance to conventional chemotherapy and radiation therapy. As a result, surgical resection is the primary therapeutic option.

#### Imaging features

**Figure 1:**
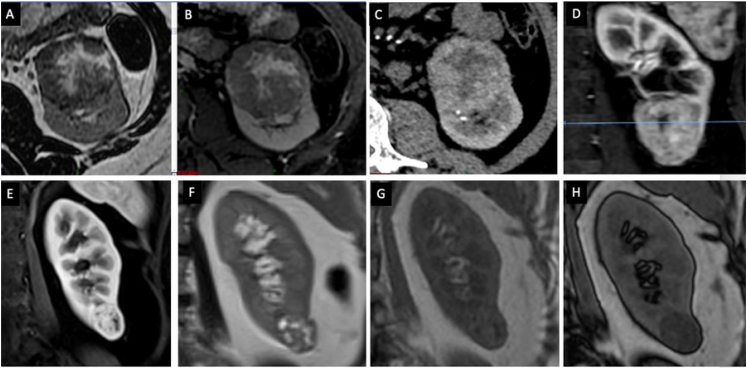
Imaging features of the clear-cell renal cell carcinoma subtype. (A, F) (T2WI), and (B) (fat-sat T2WI) show a heterogeneous lesion with liquefied or necrotic areas. (C) (CT) and (D) (magnetic resonance imaging) images show hyperenhancement in the corticomedullary phase, simillar to (E) (magnetic resonance imaging). (G) and (H) images demonstrate signal drop on opposed-phase imaging, demonstrating intralesional microscopic fat.

Clear-cell renal cell carcinoma typically appears as a well-defined, hypervascular, and heterogeneous mass that grows from the cortex in a classic "ball-type" exophytic pattern on sectional imaging exams. This growth pattern tends to displace or distort the adjacent renal parenchyma rather than invade it (Figure 1). Hypervascularity of ccRCC comes from a rich network of capillaries surrounding the tumoral cell nest. The mass may contain necrosis, calcification, or hemorrhage, contributing to its variable appearance^10^.

On MRI, the tumor shows a variable T2W signal intensity, usually hyperintense or isointense. It may present a characteristic opposed-phase signal intensity drop resulting from the high glycogen and lipid content of its "clear" cytoplasm. The mass appears hypointense or heterogeneous on T1W imaging, reflecting its hydrated or often necrotic and hemorrhagic content. DWI restriction is variable, and it typically presents a marked restriction.

### Papillary renal cell carcinoma

#### Overview

Papillary renal cell carcinoma is a type of kidney cancer that is typically well defined and can be identified by its papillary or tubulopapillary architectural patterns in the renal cortex^5^. It is the second most common subtype of RCC, accounting for approximately 13–20% of renal epithelial tumors. Although it is primarily found in adults, it can also occur in children^9^. PRCC can appear as single or multiple tumors, and it is not uncommon to appear bilaterally in patients with chronic renal disease. It is usually asymptomatic and is often detected incidentally during imaging studies. Macroscopically, pRCCs can have varying appearances, ranging from yellow to red-brown or variegated, due to factors such as hemorrhage, necrosis, foamy macrophages, cholesterol, or hemosiderin. Compared to other subtypes of RCC, such as ccRCC or unclassified RCC, pRCC generally has a more favorable prognosis.

#### Imaging features

**Figure 2:**
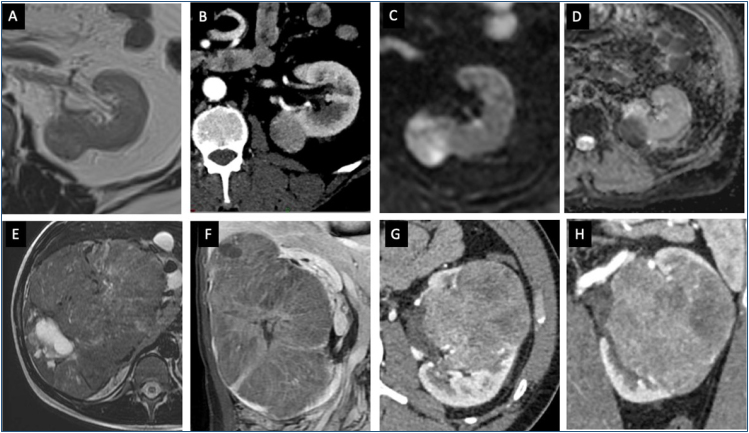
Papillary (A–D) and chromophobe RCC (E–H) features. PRCC is shown in (A) (T2WI imaging) as a homogeneous intermediate signal nodular lesion, hypovascular on (B) (CECT), and presenting marked diffusion-weighted imaging restriction (C) and low signal on the ADC map (D). Chromophobe RCC features are shown on (E–H) as a large heterogeneous lesion, hypovascular, with a central scar.

Papillary renal cell carcinoma often appears as a hypovascular or iso-vascular lesion that enhances less than the normal renal cortex and demonstrates a low signal on T2W sequences^11-13^. However, areas of hyper-vascularity can be seen, particularly at the periphery of the tumor (Figure 2). Although smaller lesions are usually homogeneous, they may display a combination of cystic and solid components, resulting in regions of low attenuation alongside enhanced solid components. Although not always present, calcifications can appear as punctate or curvilinear densities. While these imaging features can suggest pRCC, a definitive diagnosis relies on histological examination.

### Chromophobe renal cell carcinoma

#### Overview

Chromophobe renal cell carcinoma is a distinct subtype of kidney cancer originating from the collecting duct's intercalated cells. It accounts for approximately 5% of all RCCs and is usually observed in people in their sixth decade. Patients with chRCC generally have a better prognosis than those with ccRCC, as chRCC is less aggressive and has a lower risk of metastasis^14,15^.

#### Imaging features

Chromophobe renal cell carcinoma tumors appear as well-defined masses on sectional imaging scans. Enhancement is often equal to, or lower than, the renal parenchyma. The peripheral pattern of enhancement is often observed, and a central scar may be seen (Figure 2). On MRI, chRCC is usually isointense or slightly hypointense on the T1W and T2W sequences. Necrotic areas and calcifications are infrequently observed, consistent with the well-defined and often homogeneous nature of chRCC.

The differential diagnosis of chRCC includes oncocytomas, which are benign renal tumors. Due to overlapping imaging features, notably the central scar, the differential diagnosis is often challenging. However, avid contrast enhancement favors oncocytomas over chRCC.

### Other renal cell carcinoma subtypes and renal cell carcinoma not otherwise specified

Approximately 10% of RCCs are classified as subtypes such as collecting duct, medullary, tubulocystic carcinoma, and RCC not otherwise specified (NOS)^5^. These subtypes do not have specific imaging features, and a histological diagnosis should only be suggested when well-known clinical conditions are associated with them. These clinical conditions may include falciform disease (for medullary carcinoma), genetic syndromes (such as Birt-Hogg-Dubbe and oncocytomas), and chronic kidney disease (CKD) (for acquired cystic disease-associated RCC)^16^.

### Clear-cell likelihood score

Although renal mass biopsy is an option for evaluating the histological nature of renal masses in selected cases, its use remains debatable due to its invasiveness, risk of bleeding, and potential complications^17^. In addition, specific masses located in the hilar region are difficult to target effectively, and even when adequately biopsied, they still have a non-diagnostic rate^18^ of more than 10%.

In this sense, to identify potential ccRCC among indeterminate solid renal masses through imaging methods and avoid potential unnecessary biopsies, a Likert scale-based score called the clear-cell likelihood score (ccLS) has been introduced^19^. This scoring system can be applied only to MRI studies and has demonstrated good diagnostic performance, with a positive predictive value (PPV) and negative predictive value (NPV) of around 80% for cT1a ccRCC in several retrospective studies^20^.

**Figure 3:**
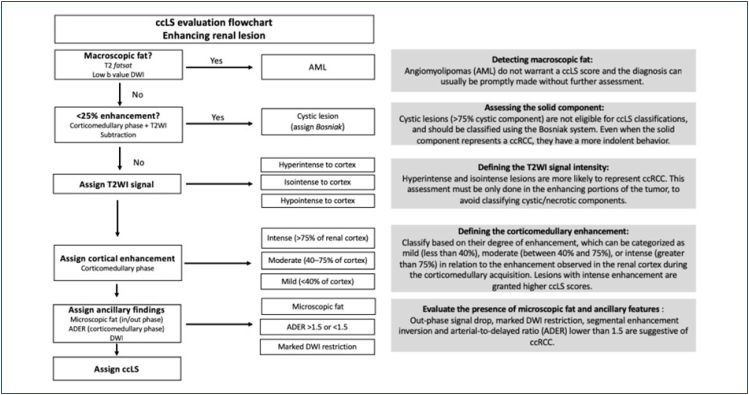
Clear-cell likelihood score evaluation flowchart. Adapted from Pedrosa et.al.^19^.

Assigning a ccLS score involves a six-step imaging assessment, as demonstrated in Figure 3.

## STAGING

### TNM staging

The tumor (T), nodes (N), and metastases (M) (TNM) system from the 8th edition of the American Joint Committee on Cancer (AJCC) is the predominant staging system for kidney cancer^21^. Radiological imaging is used to identify, classify, and determine the extent of kidney cancer. Its primary advantages are that it is non-invasive, offers precise measurement of tumor size, can visualize important landmarks for T-category assessment, and allows for the detection of pathologic lymph nodes and distant metastases. However, its limitation is that it may be unable to identify invasions into significant landmarks such as the renal capsule or Gerota fascia.

#### Tumor (T) staging

Computed tomography is the primary method for assessing the size and extent of the primary renal tumor. It effectively differentiates between tumors confined to the kidney (T1 and T2 stages) and those that extend beyond the renal capsule, either into the perinephric fat or renal veins (T3 stage). CT can also identify tumors that invade the surrounding adrenal gland or directly penetrate the ipsilateral renal fascia, indicative of the T4 stage. On the other hand, MRI provides superior soft tissue contrast and becomes particularly valuable when CT findings are unclear. MRI is excellent at visualizing tumor extensions into vascular structures like the renal vein or inferior vena cava. It is also preferred for patients who cannot undergo CT scans with iodinated contrast agents due to allergies or kidney issues.

#### Lymph node (N) staging

For RCC, regional lymph nodes primarily refer to the lymph nodes around the kidneys in the retroperitoneal space. This includes the hilar, perirenal, paracaval, and para-aortic lymph nodes. Notably, any lymph node metastasis beyond these regional nodes would be classified under distant metastasis, designated as "M1" in the TNM staging system. The probability of regional lymph node metastasis grows with the tumor's size. CT accuracy in detecting these metastases in RCC patients ranges between 72 and 99%, with a median sensitivity of 76% and specificity of 79%^22,23^. MRI has a performance comparable to CT. Both modalities struggle to distinguish between enlarged reactive and metastatic lymph nodes, and they cannot detect micrometastases in smaller nodes. While fluorine-2-fluoro-2-deoxy-d-glucose positron emission tomography/computerized tomography (FDG PET/CT) is not commonly used to stage RCC due to its subpar assessment of the primary tumor, it has a median sensitivity of 77% and a specificity of 100% in identifying lymph node metastases in RCC patients^24^.

#### Distant metastatic disease (M) staging

Renal cell carcinoma metastases can spread to any organ but most commonly affect the lungs, bones, and lymph nodes. CT and MRI scans are 85% accurate in detecting lymph node metastases^25,26^. Abdominal metastases are best detected using CT scans of the arterial and venous phases. Adrenal nodules require special attention, as it can be challenging to differentiate between benign adenomas and RCC metastases.

## SURGICAL MANAGEMENT

### Radical nephrectomy

Radical nephrectomy was the most common treatment for RCC. It involves removing the entire kidney but is associated with reduced renal function. To avoid this, NS techniques like laparoscopic and robotic-assisted PN have been developed. These methods can be expensive and require specialized training.

For less advanced RCC, NS approaches are preferred. The National Comprehensive Cancer Network (NCCN)^4^ recommends RN for Stage I-III RCC (and T1a for selected patients). The American Urological Association (AUA)^27,28^ suggests considering RNs for cases with higher oncologic potential, high tumor complexity, and normal contralateral kidney function.

### Partial nephrectomy and nephron-sparing surgery

Partial nephrectomy is now the standard treatment for small renal masses, removing the tumor while preserving the non-tumorous portion of the kidney. Studies have shown equivalent oncologic outcomes for T1 tumors between partial and radical nephrectomies.

The National Comprehensive Cancer Network^4^ recommends PN for patients with stage I–III tumors, where technically feasible, bilateral renal masses, and familial renal cell cancer. Due to its young age or medical risk factors, it is also recommended for patients at risk of developing CKD. AUA^28^ recommends PN for T1a tumors and anatomical/functional unilateral kidney, bilateral tumors, pre-existing CKD, proteinuria, multifocal masses, and comorbidities.

### Renal ablation

Ablation techniques are minimally invasive alternatives to surgical removal of tumors. They are valuable for treating small renal masses and high-risk surgical patients. Examples include radiofrequency ablation (RFA), cryoablation, microwave ablation (MWA), and high-intensity focused ultrasound (HIFU). Renal ablation is recommended for T1a and T1b tumors, especially in patients with a solitary kidney or multiple bilateral tumors associated with hereditary syndromes^29,30^.

## PREPROCEDURAL PLANNING: SCORE SYSTEMS

### R.E.N.A.L. nephrometry score

The R.E.N.A.L. nephrometry^31^ score assesses five critical attributes of renal tumors to determine their surgical complexity. It guides surgical planning and helps decide between PN and RN. The score predicts perioperative complications, longer operative times, and more significant blood loss. The R.E.N.A.L. score parameters are shown in Table 2.

**Table 2:** R.E.N.A.L and PADUA score parameters.

R.E.N.A.L nephrometry score					
	Radius (maximum diameter)	Exophytic/ endophytic	Nearness to sinus/colleting system	Anterior/ posterior	Location regarding polar lines
1 point	≤4 cm	≥50% exophytic	≥7 mm	–	Entirely above or below
2 points	4–7 cm	<50% exophytic	4–7 mm		<50% between polar lines
3 points	4–7 cm	Entirely endophytic	≤4 mm		≥50% between polar lines
**PADUA nephrometry score**					
Longitudinal (polar) location		1 point if superior/inferior/2 points if middle			Risk categories (surgical complications)
Exophytic vs. endophytic		1 point if <50% exophytic/2 points if ≥50% exophytic/3 points if entirely endophytic			
Renal rim		1 point if lateral/2 points if medial			6–7: Low risk
Renal sinus		1 point if not involved/2 points if involved			8–9: Moderate risk
Collecting system		1 point if not involved/2 points if involved			≥10: High risk
Tumor size		1 point if ≤4 cm/2 points if 4.1–7 cm/3 points if >7 cm			

### PADUA score

PADUA classification system^32^ evaluates seven anatomical features of kidney tumors (Table 2). Each feature is assigned a score, categorizing tumors based on their complexity and surgical risks.

### Ablation-focused scores: (MC)² score, P-RAC, and ablation

RENAL and PADUA scores are not effective for percutaneous ablation. Three new scoring systems have been developed: (MC)² score predicts complications after cryoablation^33,34^, the P-RAC score^35^ considers tumors near sensitive structures, and the ABLATE algorithm^36^ identifies procedural challenges. Cryoablation is recommended for tumors smaller than 3 cm. Therefore, the evaluation of the tumor's proximity to the bowel is crucial. The ABLATE algorithm suggests methods to avoid damage to adjacent structures and offers guidance based on tumor location.

### Three-dimensional reconstruction

Three-dimensional reconstruction is a valuable tool in surgical planning, creating detailed models using CT or MRI data. Accurately displaying anatomical structures and tumor morphology improves surgical planning, increases surgeon confidence, and reduces risks during surgery^37^. The main goal of 3D reconstruction is to show the relationship between the tumor and hilum structures. The 3D reconstruction helps with surgical planning and the arterial clamping approach.

## REPORTING RECOMMENDATIONS

Our recommended reporting guidelines are presented in Table 3, based on our experience and recommendations by the Society of Abdominal Radiology 2016 survey on radiologists’ and urologists’ preferences^38^.

**Table 3:** Reporting guidelines for renal mass evaluation.

Morphology		
	**General guidance**	**When to report**
Size	AP×LL×CC measures	Every report
Composition (solid/cystic)	If >75% cystic provide Bosniak	
Enhancement	Hyper, iso, or hypo compared to cortex	
Necrotic component	Provide %, if possible, to estimate	
Macroscopic fat	T2WI fat-sat or b50 or <–10 UH at CT	
Microscopic fat	In-phase/out-phase signal drop	
T2W1 signal intensity	Hyper, iso, or hypo to cortex	Provide if ccLS score is given
DWI restriction	Degree of restriction (marked)	
ADER	>1.5and <1.5	
**Location**		
Laterality	Left vs. right	Every report
Polar location	Upper vs. lower pole	
Relation to polar lines	If crosses either and % between lines	
Exophytic/endophytic component	Provide %	
Axial location	Anterior vs. posterior	
Bowel proximity	Useful for ablation	If candidate for ablation
Adjacency to ureter	Useful for ablation	
**Staging**		
Invasion of perirenal fat	Invasion vs. no invasion	Every report
Invasion/proximity with sinus fat	Invasion vs. no invasion	
Invasion/proximity with collecting system	Invasion vs. no invasion	
Invasion/proximity with venous system	Report tumoral thrombosis and extension to IVC	
Tumoral thrombosis	If present, provide length, distance to IVC, hepatic venous confluence, diaphragm, and right atrium	
Invasion of adjacent organs	Invasion vs. no invasion	
Regional lymph nodes	Provide sizes of largest ones	
Distant metastasis	Provide sizes of largest ones	
**Renal anatomic relations**		
Arterial anatomy	Detail anatomy and variations	If candidate for surgery/ablation
Renal venous anatomy	Detail anatomy and variations	
Collecting system anatomy	Cite variations	
**Scores**		
ccLS	–	Optional
R.E.N.A.L.	–	If candidate for surgery/ablation
PADUA	–	
(MC)^2^	–	If candidate for ablation
P-RAC	–	
Ablation	–	
